# *Soundgen*: An open-source tool for synthesizing nonverbal vocalizations

**DOI:** 10.3758/s13428-018-1095-7

**Published:** 2018-07-27

**Authors:** Andrey Anikin

**Affiliations:** Division of Cognitive Science, Department of Philosophy, Lund University, Box 192, SE-221 00 Lund, Sweden

**Keywords:** Nonverbal vocalizations, Animal vocalizations, Formant synthesis, Parametric synthesis, Voice synthesis, Open source, Emotion

## Abstract

Voice synthesis is a useful method for investigating the communicative role of different acoustic features. Although many text-to-speech systems are available, researchers of human nonverbal vocalizations and bioacousticians may profit from a dedicated simple tool for synthesizing and manipulating natural-sounding vocalizations. *Soundgen* (https://CRAN.R-project.org/package=soundgen) is an open-source R package that synthesizes nonverbal vocalizations based on meaningful acoustic parameters, which can be specified from the command line or in an interactive app. This tool was validated by comparing the perceived emotion, valence, arousal, and authenticity of 60 recorded human nonverbal vocalizations (screams, moans, laughs, and so on) and their approximate synthetic reproductions. Each synthetic sound was created by manually specifying only a small number of high-level control parameters, such as syllable length and a few anchors for the intonation contour. Nevertheless, the valence and arousal ratings of synthetic sounds were similar to those of the original recordings, and the authenticity ratings were comparable, maintaining parity with the originals for less complex vocalizations. Manipulating the precise acoustic characteristics of synthetic sounds may shed light on the salient predictors of emotion in the human voice. More generally, *soundgen* may prove useful for any studies that require precise control over the acoustic features of nonspeech sounds, including research on animal vocalizations and auditory perception.

An important goal for research on acoustic communication is to determine how the particular characteristics of a produced sound affect its meaning. For example, acoustic correlates of different affective states can be identified by comparing recordings that were obtained in different contexts (Briefer, 2012; Hammerschmidt & Jürgens, 2007) or that are perceived as expressing different emotions (Banse & Scherer, 1996; Sauter, Eisner, Calder, & Scott, 2010). Correlation is not causation, however: To determine which acoustic features actually influence the perceivers, methodologically the most powerful approach is to modify the signal, one feature at a time (Scherer, 2003). Speech synthesis is a diverse and mature field (Schröder, 2009), but fewer options are available to researchers who wish to synthesize or modify human nonverbal vocalizations, such as laughs and screams, or sounds produced by nonhuman animals. For instance, it would be easier to elucidate the contested role of nonlinear phenomena in pant-hoots of chimpanzees (Riede, Arcadi, & Owren, 2007) or to determine what acoustic characteristics help listeners discriminate between spontaneous and volitional laughs (Anikin & Lima, 2018; Bryant & Aktipis, 2014) if there were a simple way to synthesize these sounds and then manipulate their acoustic properties. This is the context in which *soundgen* (https://CRAN.R-project.org/package=soundgen) was developed as an open-source tool designed specifically for the manual, fully controlled synthesis and manipulation of nonverbal vocalizations.

## What is soundgen?

*Soundgen* is an open-source library that contains tools for analyzing, manipulating, and synthesizing sounds. Its main function for sound synthesis, *soundgen()*, can generate one or more syllables with voiced and unvoiced segments. The control parameters refer to acoustically transparent and perceptually meaningful characteristics such as amplitude envelope, intonation, and various aspects of voice quality. The input code is sparse, so that an entire vocalization or even multiple syllables can be created with a single short command. For example, the intonation of the entire vocalization can be specified with a few values of the fundamental frequency (*f*_0_): one at 0 ms, another at 300 ms, and a third at 1,000 ms, producing a smooth *f*_0_ contour that passes through these anchor points. Under the hood, soundgen creates a combined harmonic-noise excitation source (Erro, Sainz, Navas, & Hernaez, 2014; Gobl & Ní Chasaide, 2010; Stylianou, 2001) and then filters it to imitate the effects of the vocal tract, enhancing certain frequencies in the spectrum (formants).

**Fig. 1:**
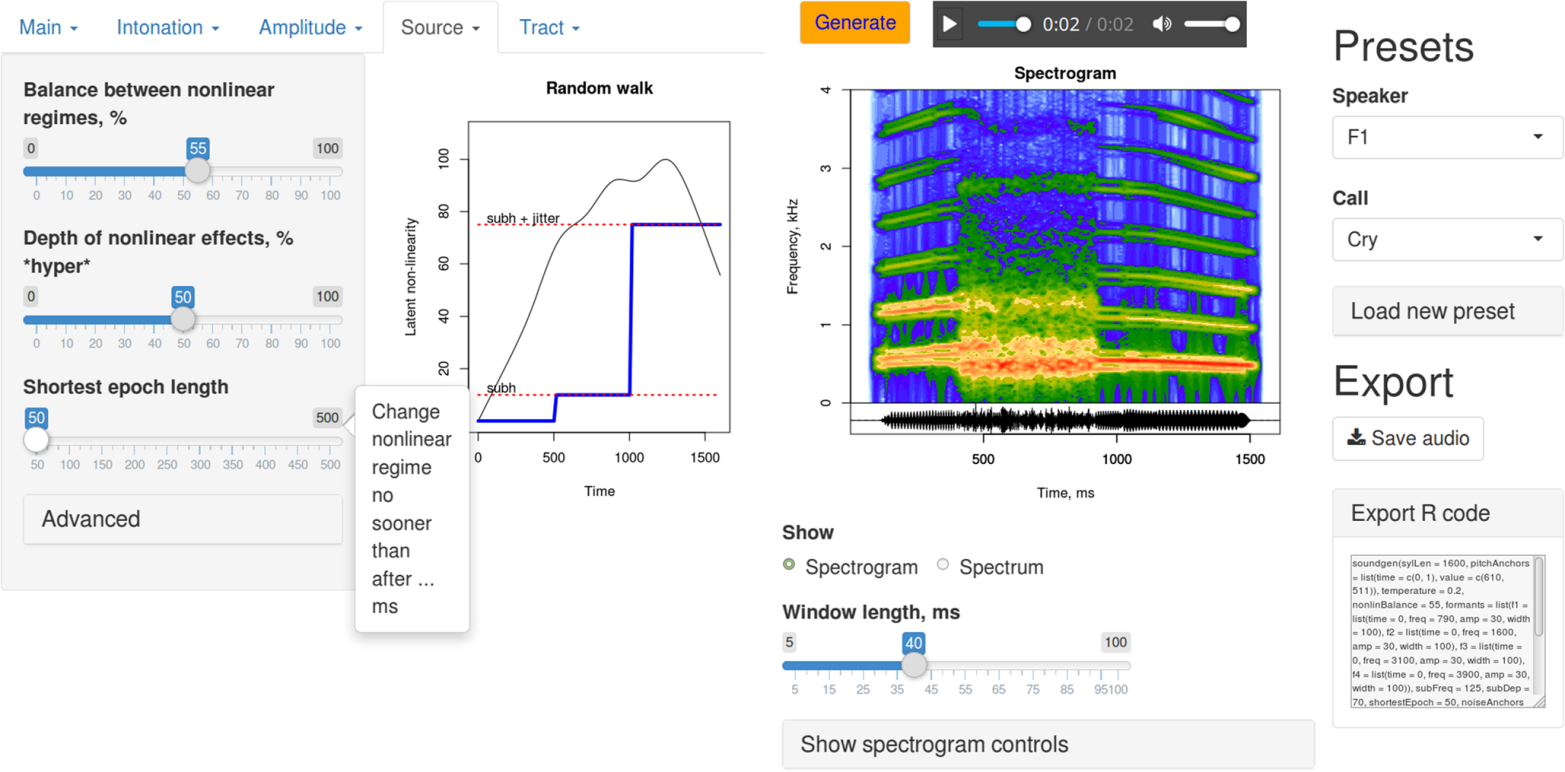
Graphical user interface for *soundgen*

*Soundgen* can be installed, used, and modified freely; it is distributed under the GPL-2/GPL-3 license as an R package available for Windows, Mac OSX, and GNU/Linux platforms. R is a popular general-purpose programming language with excellent support for sound processing thanks to a number of dedicated packages that *soundgen* imports and builds upon, particularly *tuneR* (Ligges, Krey, Mersmann, & Schnackenberg, 2016) and *seewave* (Sueur, Aubin, & Simonis, 2008). After installing R (https://www.r-project.org/), and preferably RStudio (https://www.rstudio.com), both of which are open-source, sounds can be generated from the command line using the *soundgen()* function. There is also an interactive graphical user interface (GUI), namely a Web app launched with the *soundgen_app()* function (Fig. 1).

Additional documentation is available on the project’s homepage at http://cogsci.se/soundgen.html. The so-called “vignette” on sound synthesis, which is published and regularly updated together with the package, provides an illustrated, step-by-step manual on using the *soundgen()* function. Several demos, including the R code for creating dozens of human vocalizations (laughs, roars, screams, moans, etc.) and animal sounds, are available on the project’s website. There are also numerous examples of both human and animal vocalizations built into the package itself and available through the interactive app.

## How does soundgen compare to the alternatives?

In many research applications it is not necessary to synthesize a vocalization from scratch, but simply to take an existing recording and modify some of its acoustic characteristics. Some operations are trivial: for example, the amplitude envelope of a recording can be adjusted with any audio editor. Other acoustic manipulations are more challenging and require specialized software. For instance, the effect of *f*_0_ on perceived dominance and mating preferences in humans has been investigated by manipulating *f*_0_ experimentally, often together with formant frequencies (e.g., Fraccaro et al., 2013; Puts, Gaulin, & Verdolini, 2006). However, this manipulation also affects the average distance between neighboring formants (formant dispersion), making it difficult to determine whether it was *f*_0_ or formants that caused the observed effect. It is also possible, although more technically challenging, to avoid this confound by manipulating *f*_0_ and formant dispersion independently of each other (Kawahara, Masuda-Katsuse, & De Cheveigne, 1999; Reby et al., 2005; Taylor, Reby, & McComb, 2008). For example, Reby et al. scaled formants in the roars of male red deer without changing *f*_0_ and demonstrated the important role of formant dispersion for exaggerating apparent body size during roaring contests.

Morphing is an interesting special case of manipulating existing recordings, in which certain acoustic features of sound A are gradually adjusted to match those of sound B, producing several hybrid sounds that combine characteristics of both A and B. A popular choice for such work is to use the STRAIGHT algorithm, which can morph broadly similar sounds on the basis of several manually specified landmarks that identify corresponding parts of the two spectrograms (Kawahara et al., 1999). For example, this technique was used to demonstrate categorical perception of macaque vocalizations by human listeners (Chakladar, Logothetis, & Petkov, 2008), to study acoustic features enabling individual recognition in macaques (Furuyama, Kobayasi, & Riquimaroux, 2017), and to prepare morphs of emotional vocalizations for a neuroimaging study (Salvia et al., 2014).

The techniques described above require that the researcher should prepare the stimuli in advance. For real-time manipulation, a promising tool is the *David* software (Rachman et al., 2018), which can modify the intonation and some aspects of voice quality and feed the new auditory stream back to the speaker with only a short delay. This technique was used to demonstrate that covert manipulation of a speaker’s voice suggestive of particular emotional states induces the same emotion in the speaker, as predicted by the self-perception framework (Aucouturier et al., 2016). When a slightly longer delay is acceptable—for example, for voice manipulation during interaction instead of sensorimotor feedback—a powerful technique to use is frequency warping (Erro, Navas, & Hernáez, 2013). By stretching and warping the spectral envelope on the basis of a user-defined nonlinear function, it can achieve complex manipulations of the filter in real time, for example, raising the frequencies of a few individual formants to produce the impression that the speaker is smiling (Arias et al., 2018).

The greatest advantage of sound manipulation over synthesis is that it preserves subtle characteristics of the original recording. The results can be highly naturalistic, and as long as it is feasible to achieve the desired manipulation without resynthesizing the entire sound, this is the preferred method. However, some acoustic features cannot be modified so easily (e.g., the harmonic structure of the glottal source) or require complex transformations that may degrade the quality of the manipulated sound (e.g., frequencies of individual formants). Because of these limitations, sometimes it is preferable to synthesize a vocalization de novo, which requires an algorithm for generating a waveform from a list of control parameters—a so-called “vocoder.”

Any parametric text-to-speech engine includes a vocoder, and any vocoder can potentially be used to generate nonverbal vocalizations as well as speech (e.g., Klatt & Klatt, 1990; Kreiman, Antoñanzas-Barroso, & Gerratt, 2010; Morise, Yokomori, & Ozawa, 2016). However, text-to-speech platforms usually prioritize efficiency and high-fidelity output (Tokuda et al., 2013; van den Oord et al., 2016; Wu, Watts, & King, 2016) over control of individual acoustic features (but see Birkholz, Martin, Xu, Scherbaum, & Neuschaefer-Rube, 2017; Drugman, Kane, & Gobl, 2012). As a result, they are not always optimal for academic acoustic research, particularly when working with non-speech sounds. One feature of the vocoders developed for speech synthesis is that they require a separate list of parameter values for each short frame. In other words, control parameters even for a short vocalization add up to a large matrix, which is unwieldy to work with manually. In addition, many control parameters resist simple adjustments. For example, the parameters that control voice quality do so by changing the shape of glottal pulses, and there is no simple way to predict which changes in control parameters will achieve the desired change in the output spectrum (Kreiman, Garellek, Chen, Alwan, & Gerratt, 2015).

Because of this complexity and opacity, vocoders are normally controlled by statistical models rather than manually; popular choices are hidden Markov models (HMM; Tokuda et al., 2013; Zen, Tokuda, & Black, 2009) or neural networks (Juvela et al., 2016; Ling et al., 2015; Wu et al., 2016). An even more “black-box” approach to statistical parametric synthesis is to build neural networks that directly generate raw waveforms (van den Oord et al., 2016). Whatever the exact statistical model, first it must be trained on a large annotated corpus, and the purpose of such tools is to take text as input and produce audio as output.

Text-to-speech systems are thus not the most appropriate tools if the input categories are longer than phonemes (e.g., vocalizations) or if the goal is to synthesize nondiscrete sounds (e.g., continuous modulated tones for psychophysical experiments). There have been a few attempts to synthesize nonverbal vocalizations, usually laughter, by adapting existing text-to-speech engines based on concatenative (Campbell, 2006), articulatory (Lasarcyk & Trouvain, 2007), or HMM parametric synthesis (Haddad, Cakmak, Sulír, Dupont, and Dutoit, 2016), but this research is scarce. An interesting alternative developed for resynthesis and manipulation of short verbal fragments is the UCLA Voice Synthesizer (Kreiman et al., 2010). This is an integrated analyzer–vocoder that can be analyze, reproduce, and then manipulate a recording, although it is optimized for working with speech and does not offer the researcher an easy solution for generating a completely new vocalization based on a high-level acoustic description.

Unlike integrated platforms for parametric speech synthesis, which consist of a vocoder and a statistical model to operate it, *soundgen* is essentially only a vocoder, giving the user direct access to acoustic controls. As a result, its control parameters must be kept transparent and sparse—defined at a low temporal resolution with a few anchor points. Another difference between *soundgen* and the vocoders used for text-to-speech conversion is that the latter are designed to synthesize speech, so their settings are optimized for human voice, more specifically for the voice quality typically encountered in ordinary conversation. In particular, several mathematical models can approximate the shape of glottal pulses (Fant, Liljencrants, & Lin, 1985; Rosenberg, 1971; Shue & Alwan, 2010), but they are less accurate for nonmodal phonation (Gobl & Ní Chasaide, 2003) and may not be suitable for nonverbal vocalizations such as high-pitched screams or noisy roars. It is even less certain whether the same parametric models of glottal pulses will capture the excitation source in nonhuman animals, who display a variety of sound production mechanisms, from the glottal whistles of rodents to the syringeal phonation of birds (Goller, 2016). In fact, even if a vocoder closely matches the shape of glottal pulses, it does not guarantee that the perceptually relevant spectral characteristics will be captured successfully. This is why it has been suggested that auditory perception in humans is better modeled in the frequency than time domain (Doval & d’Alessandro, 1997; Kreiman et al., 2015). Furthermore, despite all the diversity of sound sources in the animal world, the source-filter model (Fant, 1960) still holds across species (Goller, 2016; Taylor & Reby, 2010). Capitalizing on the flexibility and general applicability of spectral-domain modeling, *soundgen* creates individual harmonics instead of individual glottal pulses (it does support pulse-by-pulse synthesis, but this is not the default method), making it straightforward to directly adjust the perceptually relevant characteristics of the glottal source for any mode of phonation and potentially across species.

In this sense *soundgen* is more similar to the Mammalian Vocal Synthesizer implemented in the MiRo biomimetic robot (Moore, 2016), which specifically aims to model voices of different mammals and has inspired some of *soundgen*’s features. The main difference is that *soundgen* offers more flexible control, making it more suitable for perceptual experiments that involve precise acoustic manipulations. On the other hand, the Mammalian Vocal Synthesizer is easier to control, and it runs in real time, making it a better choice for interactive systems such as social robots.

## Implementation details

A brief summary of the fundamental principles of voice synthesis with *soundgen* is provided below. The actual control parameters are described fully from a user’s perspective in the package documentation, particularly in the so-called “vignette” on sound synthesis.

### Excitation source

Although early vocoders used a train of rectangular impulses as a source of excitation, this produced a relatively flat source spectrum with too much high-frequency energy, which made the voice sound buzzy (Cabral, Renals, Richmond, & Yamagishi, 2007). Modern vocoders solve this problem by using more realistic excitation sources that resemble actual glottal pulses produced by vibrating vocal folds (Fant et al., 1985; Klatt & Klatt, 1990). In contrast to this time-domain model of the glottal excitation source, *soundgen* generates a separate sine wave for each harmonic, covering one voiced syllable instead of one glottal pulse at a time. At each time point the frequency of each sinusoidal component is an integer multiple of *f*_0_, which can vary within one syllable. This approach is similar to tonal synthesis as implemented in the *seewave* R package and described in detail by Sueur (2018). For each harmonic *h* in {1, 2, . . .}, the waveform *w*_*h*_(*t*) for harmonic *h* is synthesized as a sine wave with time-varying frequency *h* * *f*_0_(*t*) and amplitude *a*_*h*_(*t*):

$$ {w}_h(t)={a}_h(t)\ast \mathit{\sin}\left(2\ast pi\ast h/s\ast {\sum}_1^t{f}_0(t)\right), $$

where *t* is an integer index of the synthesized point, *f*_0_(*t*) is the instantaneous fundamental frequency at time *t*, *a*_*h*_(*t*) is the instantaneous amplitude of harmonic *h* at time *t*, and *s* is the number of points per second of audio (sampling rate). The phase of each harmonic is set to zero. The final waveform is then given by the sum of all harmonics. The number of synthesized harmonics is determined by the sampling rate: no harmonics are synthesized above the Nyquist frequency (half the sampling rate) to avoid aliasing. The relative strength of harmonics relative to *f*_0_ is governed by a family of *rolloff* parameters, the most important of which gives the rate of exponential decay in the amplitude of upper harmonics: with each octave above *f*_0_, the power of harmonics decreases by *rolloff* dB. Rolloff can also be automatically adjusted depending on *f*_0_, and the source spectrum may assume more complex shapes than a simple exponent, providing more flexibility with the excitation source.

In addition to the harmonic component, *soundgen* generates broadband noise with a spectrum that is flat up to a certain threshold (by default 1200 Hz) and has an adjustable linear spectral slope of *rolloffNoise* dB/kHz in higher frequencies (Johnson, 2011; Stevens, 2000). Noise is created in the frequency domain by drawing from a uniform distribution, multiplied by the rolloff function and the vocal tract transfer function (see below), and then converted to a waveform via inverse short-time Fourier transform (STFT). The noise component is then modulated with its own amplitude envelope, which is specified independently of the amplitude envelope of the voiced component.

The manner in which the harmonic and noise components are added up depends on whether or not they should be filtered with the same vocal tract transfer function. The default behavior is to assume that the noise originates close to the glottis and passes through the same vocal tract, as when an animal is breathing. If the source of the obstruction lies further from the glottis, its filter is different from that of the harmonic component (Stevens, 2000). To synthesize such non-glottal noises as hissing, *soundgen* can handle a separate filter function for its noise component. It is also possible to synthesize voiceless sounds without any harmonic component.

### Filter

The sound changes as it passes through the vocal tract: Some frequencies, known as “formants,” are amplified, whereas other frequencies may be dampened (Fant, 1960). The spacing between formant frequencies depends on the length of the vocal tract from the source of excitation (glottis in mammals, syrinx in oscine birds) to the opening through which the air escapes. In *soundgen* an entire vocalization is first synthesized without formants, and then it is filtered through the vocal tract transfer function—a matrix that specifies a scale coefficient for each frequency bin and each time step in the spectrogram of an unfiltered waveform. This process involves taking an STFT of the generated waveform, multiplying the resulting spectrogram by the vocal tract transfer function, and then performing inverse STFT to transform the signal back to a waveform.

The transfer function is determined by time-varying frequencies, amplitudes, and bandwidths of several formants, which are either specified by the user or estimated from the length of the vocal tract. If the user provides the frequencies of the first few formants, these are used to estimate the apparent vocal tract length (VTL) using the regression method described in Reby et al. (2005). Additional formants are then added above the user-specified ones, with frequencies determined according to the uniform tube model (Stevens, 2000):

$$ {\displaystyle \begin{array}{c}{F}_n=\left({2}^{\ast }n\hbox{--} 1\right)/{2}^{\ast }d,\\ {}d=c/\left({2}^{\ast } VTL\right),\end{array}} $$

where *F*_*n*_ is the *n*th formant, *d* is formant dispersion, and *c* is the speed of sound in warm air (35,400 cm/s). If only VTL is specified, a neutral schwa [ə] with equidistant formants is produced. Formant frequencies are adjusted according to the degree of mouth opening using a formula adapted from Moore (2016):

$$ \Delta F={\left(m\hbox{--} 0.5\right)}^{\ast }\ c/\left({4}^{\ast }\ \mathrm{VTL}\right), $$

where Δ*F* is the change in formant frequency and *m* is the degree of mouth opening (0 = *closed*, 1 = *fully open*, 0.5 = *default neutral position, no adjustment*). When the mouth is completely closed, the sound is also nasalized by increasing the bandwidth of the first formant to 175 Hz and creating a new zero-pole pair in the vicinity of the first formant, as described in Hawkins and Stevens (1985). These settings are presumably specific to human voice, so it is not recommended to use the closed mouth feature for animal vocalizations; the corresponding formant transitions can be specified manually instead. The effects of sound radiation through the lips or the nose are controlled by two separate parameters instead of embedding them in the source spectrum, which makes it easy to adjust the settings for nonhuman biological sounds.

Unless specified by the user, formant bandwidths are estimated from frequency using an empirical formula derived from human phonetic research, namely the TNF-63 approximation (Tappert, Martony, & Fant, 1963) corrected below 500 Hz to increase bandwidth at low frequencies (Khodai-Joopari & Clermont, 2002). Once time-varying formant frequencies and bandwidths have been determined, the vocal tract transfer function is calculated in the frequency domain by using a standard all-pole model if there are only formants, or a zero-pole model if there are also antiformants (Stevens, 2000). The only modification of these models in *soundgen* was to enable more flexible control over the strength of individual formants. Mathematical details of this algorithm are beyond the scope of the present article; the relevant code can be found in the function *getSpectralEnvelope()*.

### Other control parameters

Both the source of excitation and the filter can be modified in many ways using a number of control parameters, some of which are mentioned below. Most of these parameters are vectorized, so that the amount and quality of each effect can vary over time. A vibrato can be added as a sinusoidal modulation of *f*_0_ with adjustable frequency and depth. Variation in *f*_0_ can also be stochastic (jitter), again with time-varying depth and frequency—from slow, vibrato-like random fluctuations to very rapid pitch jumps that can be used to simulate harsh voices in roars and noisy screams. Attack at the beginning and end of voiced fragments can be specified separately from the overall amplitude envelope, and rapid stochastic amplitude modulation can be added to simulate pulse-to-pulse variation in glottal pulses (shimmer). Low-frequency amplitude modulation with adjustable depth, frequency, and shape is useful for making trill-like sounds.

A special subroutine in *soundgen* is devoted to nonlinear effects, namely subharmonics (or sidebands) and deterministic chaos (Wilden, Herzel, Peters, & Tembrock, 1998). Subharmonics are created by generating additional harmonics in the excitation source, which corresponds to introducing an additional fundamental frequency (*g*_0_) at an integer ratio to *f*_0_. Chaos is simulated by adding strong jitter and shimmer. The parts of vocalization affected by nonlinear phenomena can be specified explicitly by the user or determined stochastically. A random walk is generated; its bottom part corresponds to fragments with no nonlinear effects, the middle part to subharmonics, and the highest part to both subharmonics and chaos (cf. Fitch, Neubauer, & Herzel, 2002). This makes it possible to generate sounds with unpredictable transitions between different regimes of nonlinear phenomena.

*Soundgen* contains a number of high-level hyperparameters that affect multiple acoustic features at once. For example, *f*_0_ and formant frequencies can be adjusted in a coordinated manner with the *maleFemale* parameter. The most important hyperparameter, *temperature*, adjusts the amount of stochastic variation in *f*_0_ contour, voice quality, and most other control parameters. A natural vocalization is seldom completely static, and this stochastic behavior offers an easy way to introduce some variability without manually coding every irregularity. If *temperature* is above zero, calling the *soundgen()* function repeatedly with the same settings does not produce identical output every time. This is helpful when the purpose is to create a number of authentic-sounding and similar, but not identical, vocalizations. When the goal is to generate a sound with high precision (e.g., when synthesizing multiple modifications of the same basic vocalization for perceptual testing), stochastic behavior is not desirable, and *temperature* should be set to a small positive value (setting it to exactly zero disables the addition of new formants above the user-specified ones and is not recommended).

## Validation experiment

To validate *soundgen* as a tool for synthesizing human nonverbal vocalizations, a number of laughs, screams, moans, and other sounds were synthesized aiming to approximately reproduce the original recordings. The similarity of the synthetic stimuli to their originals was then assessed in a perceptual experiment by means of comparing their ratings on three continuous scales—valence, arousal, and authenticity—as well as their classification by emotion.

### Method

#### Stimuli

Sixty authentic human nonlinguistic vocalizations were chosen from a previously published corpus (Anikin & Persson, 2017) and reproduced with *soundgen* 1.1.1. The selection criteria were (1) a minimum amount of background noise, echo, clipping, or other acoustic impurities, (2) a high degree of consensus on what acoustic type (laugh, scream, and so on) the sound represented in a previous cross-linguistic naming study (Anikin, Bååth, & Persson, 2018), and (3) high perceived authenticity, as reported by Anikin and Lima (2018). To constrain the acoustic complexity of the stimuli, longer bouts were truncated. The average duration was 1.5 ± 0.7 s (*M* ± *SD*), range 0.3 to 3.4 s (Table 1). All sounds were then normalized for peak amplitude and down-sampled to a rate of 22050 Hz.

**Table 1:** Characteristics of the original recordings in Experiment 1

Acoustic type	Number of sounds (M/F)	Duration, s Mean [range]
Cry	10 (4/6)	2.1 [1.3, 2.7]
Gasp	5 (2 /3)	1.6 [1.1, 2.6]
Grunt	5 (3/2)	0.5 [0.3, 0.8]
Laugh	10 (5/5)	1.9 [1.0, 3.4]
Moan	10 (5/5)	1.7 [0.7, 3.1]
Roar	10 (6/4)	1.4 [0.6, 3.2]
Scream	10 (2/8)	1.4 [0.4, 3]
TOTAL	60 (27/33)	1.5 [0.3, 3.4]

Control parameters were chosen manually, on the basis of an iterative visual comparison of the spectrogram with the target. The difficulty of this task varied depending on the complexity of the target, from a few minutes of work for simple moans or screams to a few hours for some laughs. Whenever possible, the entire sound was synthesized with a single command (see Table 2 for an annotated example). Some of the more complex polysyllabic vocalizations were synthesized one segment at a time and then concatenated. The *temperature* parameter was usually kept positive—that is, most sounds were synthesized in a stochastic mode. For example, for polysyllabic vocalizations the length of syllables and pauses between them was deliberately allowed to vary at random and deviate from the original. The synthetic sounds were thus not meant to be exact replicas of the originals, but only approximations. Highly stochastic sounds, such as screams consisting of segments with various vocal regimes (tonal, subharmonics, deterministic chaos) were generated several times with the same settings, and the copy closest to the original was retained for testing.

**Table 2:**
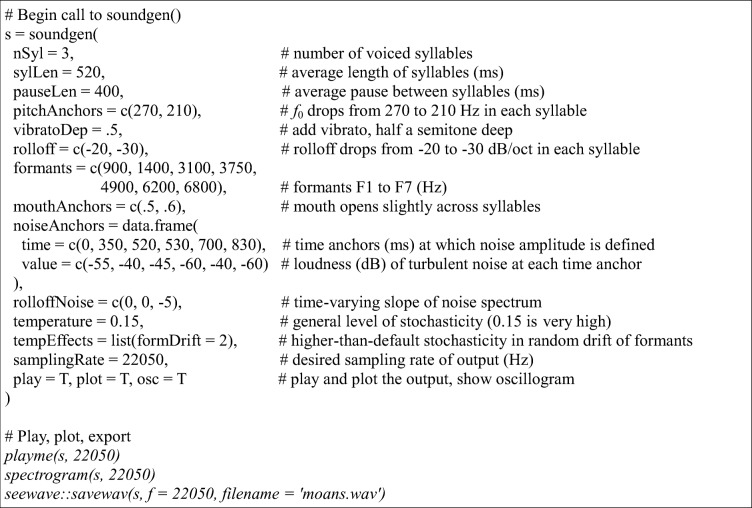
R code for generating moaning in *soundgen* version 1.1.1 and above (stimulus #38)

For more examples, see demos on the project’s homepage: http://cogsci.se/soundgen.html

Supplementary materials for this article, including all sound files, R code for their generation, raw data from the validation experiment, and R scripts for statistical analyses, can be downloaded from http://cogsci.se/publications/anikin_2018_sova.html. Note that *soundgen* has been extensively upgraded since the time of the validation experiment. It may therefore be preferable to use the more up-to-date demos from the project’s website, rather than the code from the supplementary materials, as templates for sound synthesis.

#### Procedure

The validation experiment included three separate tasks, which were performed by different groups of participants. In Tasks 1 and 2, participants rated 60 synthetic (Task 1) or real (Task 2) vocalizations on three scales: valence (“How pleasant or unpleasant is the experience?”), arousal (“How high is the level of energy, alertness?”), and authenticity (“Does the person sound natural, like in real life?”). Prior to testing, each participant was informed which type of sounds, human or synthetic, they would hear, ensuring that authenticity ratings would not be interpreted as binary guessing between “real” and “fake” sounds. To minimize the correlation between ratings on the three scales, only one scale was displayed in each block. Each sound was thus presented three times. The order of blocks and of sounds within each block was randomized for each participant.

In Task 3, participants indicated the emotion portrayed by each sound and rated their confidence in this classification. There were ten emotional categories to choose from: *amusement*, *anger*, *disgust*, *effort*, *fear, pain*, *pleasure*, *sadness*, *surprise*, and *other/neutral/don’t know*. To avoid presenting the same sound twice (the original and the synthetic version), this task was split into two subtasks, each with 60 unique sounds (30 human + 30 synthetic). Each subtask was performed by a different sample of participants. Sounds could be repeated as many times as needed. The average completion time was 10–15 min.

#### Participants

Out of the 106 participants included in the final analysis, 47 were volunteers contacted via online advertisements, and 59 were recruited via https://www.prolific.ac and received £1 or £1.5, depending on the task. An informal comparison of the data from volunteers and paid participants revealed no systematic differences, and their responses were pooled. Each participant performed only one task. The numbers of responses per sound were as follows: ratings of synthetic sounds 19.6 (range 19.0 to 20.3), ratings of human sounds 21.7 (range 21.0 to 22.0), forced choice classification of emotion for mixed human and synthetic sounds 30.7 (range 28.0 to 35.0).

Data were collected via the Internet. Participants were informed about the goal of the study and agreed with the conditions of confidentiality by clicking the active link after reading the instructions. No personal or demographic information was collected. Online experiments are increasingly being used for academic research (Hewson, Vogel, & Laurent, 2016), but the responses may be noisy compared to those from face-to-face testing. To ensure data quality, all submissions were first manually checked for fraud (e.g., clicking through stimuli very fast and without varying the responses). Once data collection was complete, a second round of verification was performed by means of correlating the ratings provided by each participant with the median ratings per stimulus aggregated from all responses. Participants were excluded from the analysis on the basis of the following criteria: (1) correlation with global median < 0.3 on any two scales, or (2) correlation with global median < 0 on either valence or arousal scale, or (3) proportion of emotion classification corresponding to the most commonly chosen emotion per stimulus < 0.3. This identified five participants, typically with very short response times, who were removed from further analysis. In addition, all trials with response time under 1 s (0.4% of the data) were excluded, since they presumably represented technical glitches.

#### Statistical analysis

Except when otherwise stated, all analyses were performed on unaggregated, trial-level data using mixed models. To account for non-independence of observations, all models included random intercepts per participant and per stimulus. Ratings on continuous scales were not normally distributed, and they were modeled with beta distributions. Bayesian models were created in Stan computational framework (http://mc-stan.org/) accessed with *brms* package (Bürkner, 2017). To improve convergence and guard against overfitting, mildly informative regularizing priors were used for all regression coefficients. Fitted values are reported as the median of the posterior distribution and 95% credible interval (CI). Intraclass correlation coefficients were calculated with the *ICC* package (Wolak, Fairbairn, & Paulsen, 2012).

### Results

#### Valence, arousal, and authenticity ratings

**Fig. 2:**
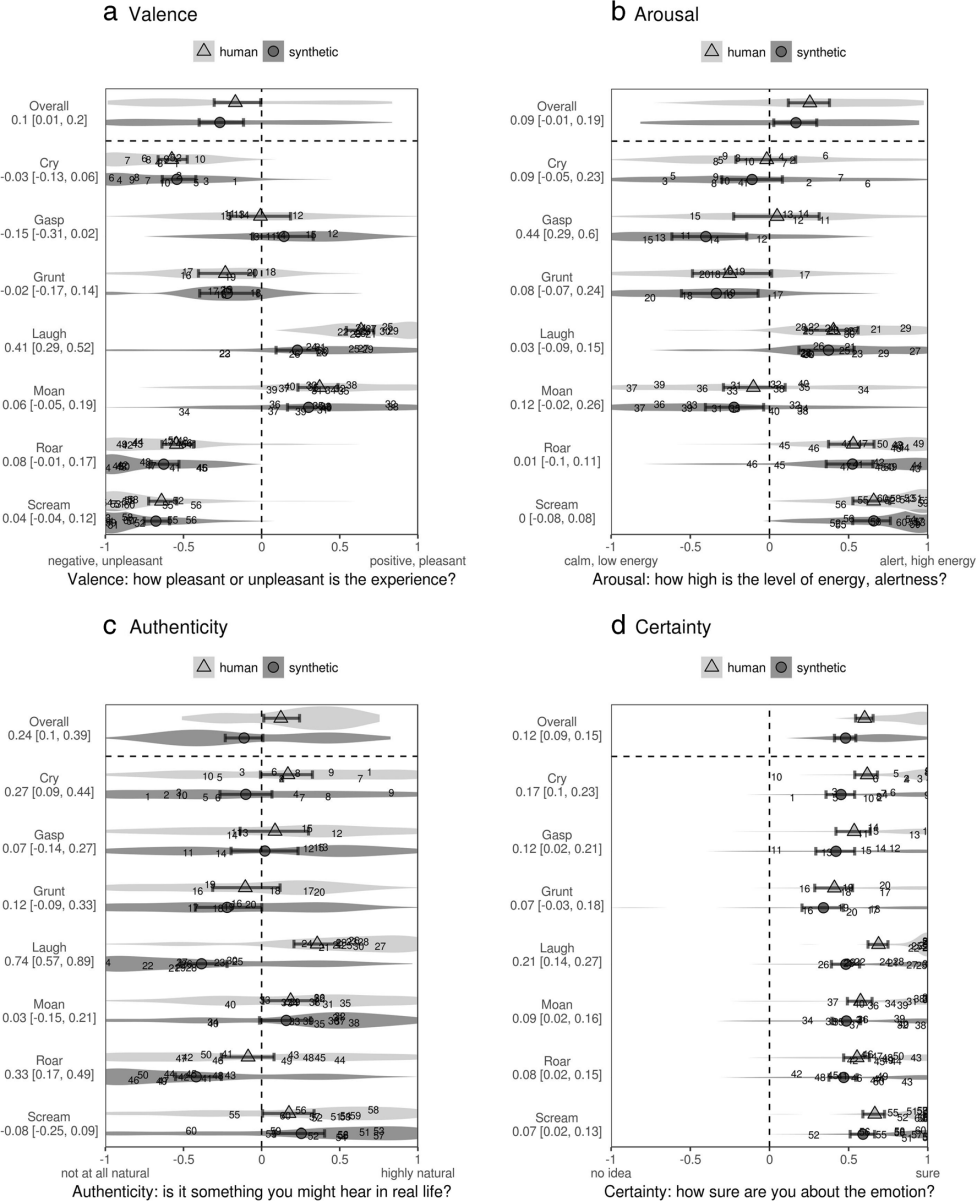
Ratings of 60 human and 60 synthetic nonlinguistic vocalizations. Violin plots show the distributions of individual ratings for each call type (the “overall” category is aggregated per stimulus), with individual stimuli marked by indices from 1 to 60. Solid points with error bars show fitted values per call type: the median of the posterior distribution with 95% CI. Contrasts between real and synthetic sounds per call type are shown as axis labels

The reliability of valence ratings was moderate: The intraclass correlation coefficients (ICCs) were .61 for human sounds and .54 for synthetic sounds. The average per-stimulus valences of human and synthetic sounds were highly correlated: Pearson’s *r* = .88, *F*(1, 58) = 207.1, *p* < .001. A more nuanced analysis with mixed models revealed that human and synthetic sounds were rated similarly on the valence scale for all call types except laughter (Fig. 2A), for which the real recordings were judged to be more positive than the synthetic sounds: by + 0.41 on a scale of – 1 to + 1, 95% CI [0.29, 0.52].

The consistency with which participants used the arousal scale was again moderate: ICCs = .42 for human and .55 for synthetic sounds. The average arousal ratings of human and synthetic sounds were highly correlated: *r* = .92, *F*(1, 58) = 327.8, *p* < .001. There was a slight tendency for human sounds to have higher arousal ratings than the synthetic sounds for several call types (Fig. 2B), but the difference was statistically significant only for gasps: + 0.44, 95% CI [0.29, 0.60]. Interestingly, the valence and arousal scales were not completely orthogonal. Averaging per stimulus, there was a quadratic relationship between valence and arousal ratings, *F*(2, 117) = 43.4, *p* < .001, *R*^2^ = .43: Sounds with either very positive or very negative valence tended to have high arousal ratings.

There was little agreement among participants about the authenticity of individual sounds, with ICCs of only .15 for human sounds and .27 for synthetic sounds. The correlation between the average authenticity ratings for human and synthetic sounds was also weaker than in the case of valence and arousal ratings, although it was still significantly higher than would be expected by chance: Pearson’s *r* = .33, *F*(1, 58) = 6.9, *p* = .01. As expected, the real recordings were overall judged to be more authentic than the computer-generated sounds: + .22 on a scale of – 1 to + 1, 95% CI [.07, .36]. It is worth emphasizing that before the experiment, participants were informed which type of sounds, human or synthetic, they would hear. Nevertheless, the difference in authenticity was statistically significant only for laughs (.74 [.57, .89]), roars (.33 [.17, .49]), and cries (.27 [.09, .44]), but not for the remaining four call types (Fig. 2C). In fact, the average authenticity of 22 out of the 60 synthetic sounds was higher than the authenticity of the original recordings.

Considering the experimental nature of the algorithms used for adding subharmonics and chaos, it was important to check the authenticity of sounds containing these nonlinear vocal phenomena. Out of 60 stimuli, six were synthesized with pitch jumps, another six with subharmonics, two with biphonation (the originals contained ingressive whistles), 23 with chaos, and 23 without nonlinear effects. The differences in authenticity between the real and synthetic versions were similar for sounds with subharmonics versus no nonlinear effects (.12 [– .05, .28], on a scale of – 1 to + 1), chaos versus no nonlinear effects (– .09 [– .19, .01]; a marginal advantage for sounds with chaos), and pitch jumps versus no nonlinear effects (.03 [– .13, .19]). More stimuli would need to be synthesized to test this further, but at least there was no obvious disadvantage of synthetic sounds with nonlinear effects, in terms of their perceived authenticity.

#### Recognition of emotion

A new sample of participants classified a mixture of the same 60 real and 60 synthetic sounds by emotion. The stated certainty of this classification was high for all sounds (Fig. 2D), but it was .12 (95% CI [.09, .15]) higher for the original recordings than for their synthetic reproductions. This difference was small but consistent across most call types. Synthetic sounds were also slightly more likely to be placed in the residual category of *Other/neutral/don’t know*: In 19.1% of trials for synthetic sounds, and only 6.6% for human sounds, predicted difference = 12.4%, 95% CI [10.3, 14.6]. The normalized Shannon entropy of the counts of emotional labels applied to a particular sound (hereafter, “emotion vectors”; see Anikin et al., 2018) was 14.4% (95% CI [7.5, 21.0]) higher, on a scale of 0% to 100%, for synthetic versus human sounds. This suggests that there was slightly less agreement among the listeners about the emotion portrayed by synthetic sounds than with the original recordings.

**Fig. 3:**
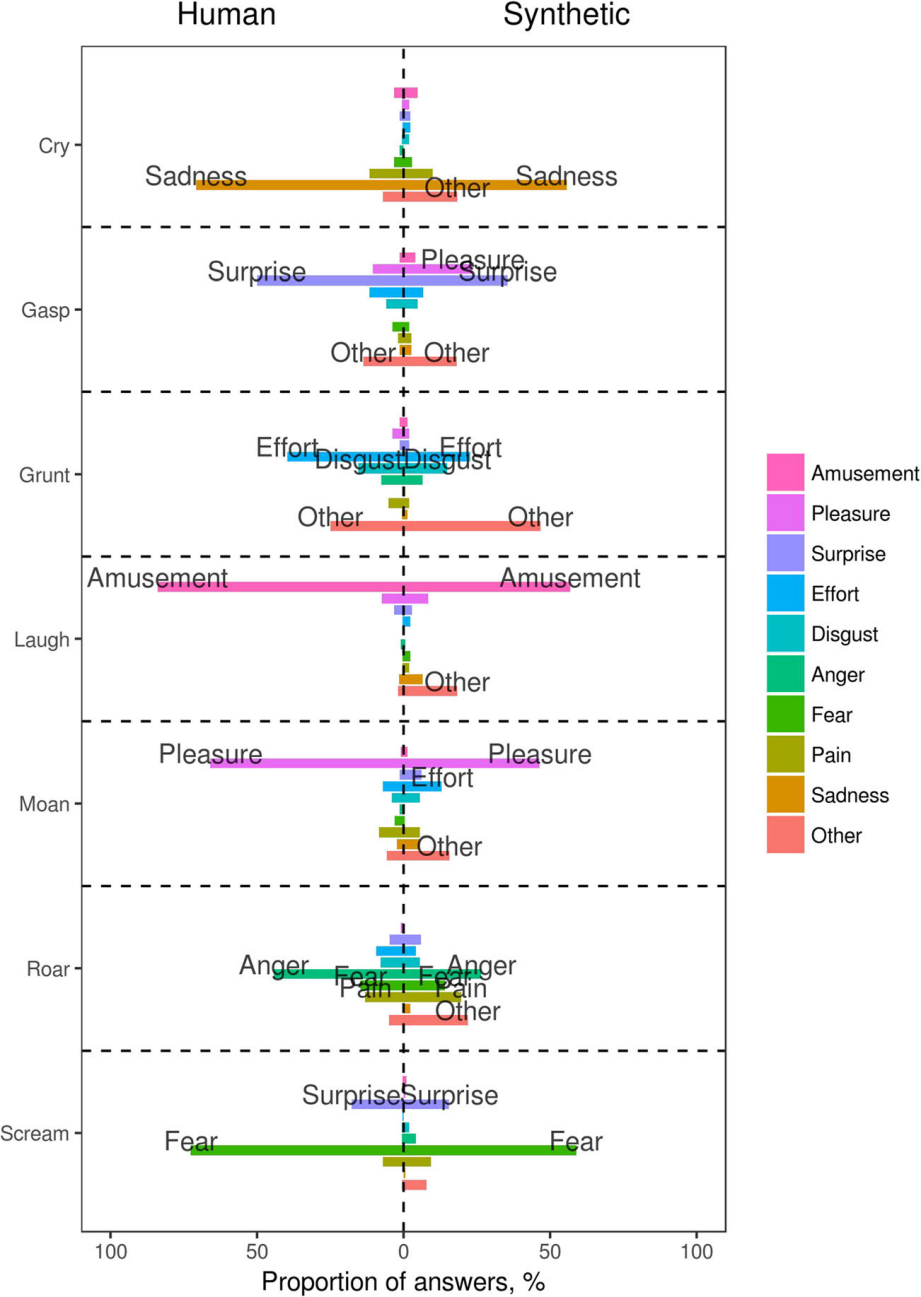
Forced choice classification of sounds in terms of their underlying emotion: Proportions of responses averaged per call type. Assuming that the synthetic versions are functionally equivalent to the original recordings, the two halves of the figure should be mirror images of each other. All bars over 12% high are labeled, to simplify reading the graph

**Fig. 4:**
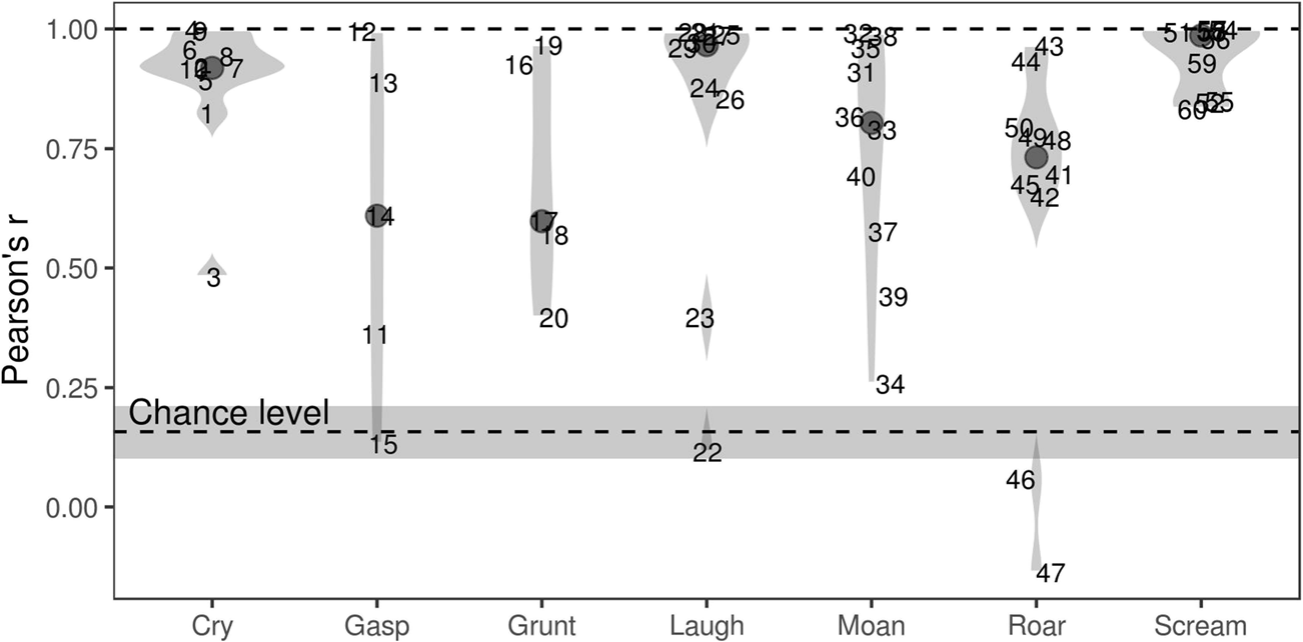
Pearson’s correlations between emotion vectors (counts of emotional labels applied to a particular sound) for real and synthetic vocalizations. Solid points mark the median for each call type, and violin plots show the distribution of values for individual stimuli, which are marked 1 to 60. The shaded area shows the correlation that would be expected by chance (median and 95% CI), which was estimated by permuting the dataset

The emotions associated with each call type were broadly similar for both real and synthetic vocalizations (Fig. 3). Looking at individual stimuli rather than call types, the correlation between the matrices of classification counts for real and synthetic sounds was high: *r* = .82, *χ*^2^(531) = 4,644.5, *p* < .001. Common measures of interrater agreement, such as Fleiss’s kappa, were not strictly appropriate: Although this was a forced choice classification task, the categories overlapped semantically and were not exclusive. For example, if laughs were classified as *amusement* by some participants and *pleasure* by others (as, indeed, sometimes happened), this might not be a sign of genuine disagreement among the raters, but kappa would drop. A more appropriate method might be to compare the classifications of each real and synthetic sound by correlating their emotion vectors. Correlations close to 1 would indicate that the distributions of responses were nearly identical for the original and synthetic versions of a particular sound, whereas low correlation would indicate systematic differences in the ways these sounds were categorized by participants. The average observed correlation between the emotion vectors of human and synthetic sounds was high (*r* = .77), as compared to the correlation expected by chance (*r* = .16, estimated by permutation). As is shown in Fig. 4, the correlations of emotion vectors were over .75 for almost all cries, laughs, and screams, but they were more variable in the remaining four call types, suggesting that a few synthetic stimuli differed from the original recordings in terms of perceived emotion.

### Discussion of the validation experiment

The validation experiment was designed to investigate whether parametric voice synthesis, as implemented in the open-source R library *soundgen*, is sufficiently flexible and precise to reproduce human nonverbal vocalizations in such a way that the perceived valence, arousal, authenticity, and emotion of the synthetic versions would be similar to those of the original recordings. It must be reiterated that synthetic sounds were not exact replicas of the originals, but stochastic generative models that aimed to preserve only the most salient and easily identifiable acoustic characteristics of the original. Even so, valence and arousal ratings of human and synthetic sounds were tightly correlated, demonstrating that the perceived affective meaning of synthetic vocalizations was very close to that of the original recordings. More importantly, synthetic vocalizations covered the entire available range on valence and arousal scales and were rated as consistently as the original human recordings. The validation study thus demonstrated that all kinds of human nonverbal vocalizations—high- or low-intensity, hedonistic or aversive—can potentially be synthesized with *soundgen*.

In addition to ensuring that synthetic sounds were close to the originals in terms of the perceived valence and arousal of the speaker, it was important to ascertain that they sounded natural and not too machine-like. Participants in the validation study were told beforehand whether they would hear human or synthetic vocalizations and then had to rate them on naturalness (authenticity). The motivation for this design was the need to evaluate the relative authenticity of both human and synthetic sounds without making the task into an attempt to guess which sounds were synthetic and which were not—this would not be particularly meaningful, since many recordings contain extraneous clues to their nonsynthetic nature (traces of background noise, a slight echo, and so on). In addition, the aim was to synthesize natural-sounding vocalizations, not to trick the listeners into believing them to be real, which is also the established practices when testing the naturalness of synthetic speech (e.g., Erro et al., 2014; van den Oord et al., 2016).

In line with previous reports (Bänziger, Mortillaro, & Scherer, 2012; Lima, Castro, & Scott, 2013), authenticity ratings were highly variable for both human and synthetic sounds and presumably depended on how often similar sounds occurred in everyday life, how genuine the speaker’s emotion appeared to be, and (for synthetic sounds) how convincing or “human-like” they sounded. Given the diversity of factors that may have affected authenticity rating of individual stimuli, they are not easy to interpret in themselves—the key metric is the difference in perceived authenticity within each pair of real and synthetic sounds. As it turned out, recordings of real people had a 10% advantage in terms of authenticity, but this difference strongly depended on the acoustic type: It was pronounced for laughs, moderate for cries and roars, and absent for gasps, grunts, moans, and screams. In other words, it was hardest to succeed in synthesizing the most acoustically complex, polysyllabic vocalizations such as cries and laughs. These vocalizations contain a rich gamut of unvoiced sounds and physiological noises (snuffling, spluttering, gurgling, wheezing, etc.), rapid formant transitions, episodes of biphonation, and a variety of transients that make it difficult for the operator to read the relevant acoustic parameters off a spectrogram and to control the synthesis manually. In addition, laughs and cries often contain a variety of syllables—they are not at all as repetitive as suggested by the conventional *Ha-ha-ha*. As a result, they have to be painstakingly synthesized in multiple steps, sometimes one syllable or even one acoustic “layer” at a time, which is time-consuming for the researcher.

Despite their lower authenticity ratings compared to the real recordings, most synthetic laughs and cries were readily recognized and correctly labeled in terms of the underlying emotion. It is therefore still possible to synthesize them, particularly if only the most authentic-sounding stimuli are retained for testing. Nevertheless, acoustically simpler vocalizations, such as moans and screams, represent much easier targets for synthesis with *soundgen*. Most synthetic vocalizations of these call types were judged to be highly authentic by the raters, although in a few cases the emotion they expressed was different from the emotion expressed by the original recording. This may partly be explained by the inherently ambiguous nature of such vocalizations as moans, grunts, and gasps (Anikin et al., 2018). In fact, the consistency with which only human (not synthetic) sounds were classified by emotion also varied across call types: The correlation of emotion vectors estimated by permutation was on average high (*r* > .95) for laughs, cries, and screams, whereas for gasps, grunts, and roars it was lower (*r* ~ .80). When there is no obvious emotion category to which to assign a sound, responses become noisier, so it is less likely that the classification decisions will be exactly the same for an original recording and its synthetic version.

There is also a second potential explanation for differences in emotional classification of certain real and synthetic sounds with high authenticity ratings. Since vocalizations like grunts and moans can potentially express a wide range of meanings, even relatively minor acoustic variations might suffice to shift the interpretation from one emotion to another. For example, Gasps 11 and 15 both had low correlations of emotion vectors between the original and synthetic versions (Fig. 2C), but for entirely different reasons. Gasp 11 had a low authenticity rating and a higher proportion of *Don’t know* responses than did the original recording, indicating that it was not synthesized very successfully. In contrast, the synthetic Gasp 15 had above-average authenticity ratings and was consistently classified as *Pleasure*, whereas the original recording was variously classified as *Surprise*, *Don’t know*, or *Effort* (the actual emotion expressed by the speaker was pleasant surprise). Subtle acoustic changes may thus suffice to cause a considerable change in the meaning of an inherently ambiguous vocalization, and identifying the responsible acoustic characteristics is exactly the kind of task that *soundgen* was designed for.

Taken together, the results of the validation experiment suggest that most of the synthetic sounds preserved the essential acoustic characteristics of the original recordings to the extent that listeners exposed to human and synthetic sounds drew the same inferences about the mental state of the caller, as measured by either continuous (valence, arousal) or categorical (emotion) outcomes. However, the most successful synthetic stimuli in the validation experiment were relatively short and simple, whereas complex and polysyllabic vocalizations, such as bouts of laughing or crying, were often rated as less natural, demonstrating that there are limits to the level of complexity that *soundgen* (or its operator) can handle.

## Suggested applications

*Soundgen* is not designed for text-to-speech conversion. Speech consists of very rapid, highly variable formant transitions and amplitude modulation. It is too complex to encode more than a few phonemes manually, which is why statistical modeling of the parameters controlling the vocoder is predominant in parametric text-to-speech systems (Schröder, 2009). However, *soundgen*’s explicit manual control becomes more appealing when the target vocalization is short or repetitive, since a reasonable synthetic approximation can be created rapidly, without having an annotated training corpus and without recalibrating the algorithm for each new species. More specifically, *soundgen* can be useful for the following applications:

1. *Synthesis of human nonverbal vocalizations, as in the validation experiment*. Naturally, the mission of parametric voice synthesis is not simply to recreate existing recordings, but to modify them in systematic ways or to generate novel sounds with desired acoustic properties (e.g., Gobl & Ní Chasaide, 2003). To explore some of these possibilities, in a follow-up study (Anikin, 2018) soundgen was used to manipulate two acoustic features that could previously be analyzed only indirectly—nonlinear vocal phenomena and the rolloff of harmonics in the source spectrum. This study provided the first evidence that perceptual effects of nonlinear phenomena depended on their type (subharmonics, chaos, or pitch jumps) and on the type of vocalization in which they occurred. It also shed new light on the effect of source spectrum on the perceived level of arousal and, for relatively ambiguous vocalizations, their valence. Such acoustic manipulations would be difficult, if not impossible, to perform without resynthesizing the sound. It would be equally difficult to elucidate the perceptual effects of such relatively subtle acoustic features on the basis of a traditional acoustic analysis of recorded vocalizations. In the future, it will also be interesting to test the perceptual consequences of manipulating other acoustic features that have previously been reported to correlate with affective states and intentions: intonation (Banse & Scherer, 1996; Ohala, 1984; Schröder, Cowie, Douglas-Cowie, Westerdijk, & Gielen, 2001), formant frequencies and transitions (Puts et al., 2006; Reby et al., 2005; Wood, Martin, & Niedenthal, 2017), the duration and number of syllables (Briefer, 2012), and many others. Moreover, synthetic vocalizations can easily be morphed by interpolating between values of control parameters manually or with the help of the built-in *morph()* function, which makes it straightforward to create series of graded stimuli, test for categorical perception, and so on.
2. *Synthesis of animal vocalizations*. Although not yet formally tested, *soundgen* should be capable of creating high-quality synthetic versions of animal calls. In fact, many design features of *soundgen* (focus on nonlinear effects, excitation source defined in the frequency rather than time domain, etc.) were specifically intended to make the algorithm generalizable to nonhuman sounds. Sine-wave synthesis has been used to produce mammalian (DiMattina & Wang, 2006; Snowdon & Pola, 1978) and avian (Margoliash, 1983) calls since the 1970s, but without specialized software only simple, pure-tone vocalizations could be created. *Soundgen*’s closest modern relative is the Mammalian Vocal Synthesizer (Moore, 2016), which is intended for real-time generation of biologically plausible sounds in a biomimetic robot. Some examples of animal calls synthesized with *soundgen* are included in the preset library published with the package.
3. *Synthesis of voice-like stimuli for psychophysical experiments*. *Soundgen* may prove useful for research on psychophysics, auditory perception, and cross-modal associations, since it offers a straightforward way to create acoustically complex sounds with precisely controlled temporal and spectral characteristics. For example, it was used to create the stimuli for research on cross-modal sound-color correspondences that required finely controlled acoustic manipulations, such as generating formant transitions without modifying the spectral centroid of synthetic vowels (Anikin & Johansson, 2018).
4. *Teaching of phonetics and bioacoustics*. The interactive app (Fig. 1) offers the advantage of immediately hearing and visualizing the effects of modifying individual acoustic features such as the glottal source, frequencies and bandwidths of individual formants, various nonlinear effects, and other acoustic features that are easier to understand with an interactive demonstration. This functionality of *soundgen* can be valuable in an educational setting.
5. *Integration with text-to-speech platforms, use in human–machine interaction*. Although suboptimal for synthesizing speech as such, *soundgen* or some of its subroutines can be adapted for introducing simple nonverbal vocalizations into speech synthesized with another engine. One way of adapting *soundgen* for this purpose would be to compile a library of presents for several common vocalizations, such as laughing or sighing, and to vary them on the basis of simple rules for modifying the acoustic parameters in accordance with the desired level of emotion intensity and valence. Nonverbal vocalizations as well as artificial, nonbiological sounds (Read & Belpaeme, 2016) are particularly interesting in the context of social robotics, since they have the potential to enrich interaction without an “uncanny valley” effect. *Soundgen* is an open-source project, so the relevant code can be easily adapted or translated to fit another platform, potentially facilitating the development of other tools. However, the principle of continuous sine-wave synthesis makes *soundgen* less suitable for the real-time generation of continuously concatenated short fragments than are vocoders that generate individual glottal pulses. In addition, *soundgen* is currently somewhat slower than real time (the exact speed depends on the nature of the synthesized sound). Computational efficiency would have to be improved for it to be used in interactive systems, such as social robots, but this slowness is not a drawback when the goal is to synthesize stimuli for testing.
6. *Creation of special sound effects*. *Soundgen* is primarily developed as a research tool, but it can be adapted for the task of creating a wide variety of nonrepetitive, parametrically controlled human and animal sounds, which can potentially be of interest for the gaming and entertainment industry.
